# Tabanidae (Diptera) of Maranhão state, Brazil. V. Description of *Protosilvius gurupi* sp. n. (Pangoniinae, Pangoniini) and key to *Protosilvius* species

**DOI:** 10.3897/zookeys.235.3786

**Published:** 2012-10-31

**Authors:** José Albertino Rafael, Dayse Willkenia Almeida Marques, Francisco Limeira-de-Oliveira

**Affiliations:** 1 Instituto Nacional de Pesquisas da Amazônia, Caixa Postal 2223, 69080–971 Manaus, Amazonas, Brazil; 2 Universidade Estadual do Maranhão, graduanda do Curso de Ciências Biológicas, 65604–380 Caxias, Maranhão, Brazil; 3 Universidade Estadual do Maranhão, Laboratório de Estudos dos Invertebrados, 65604–380 Caxias, Maranhão, Brazil

**Keywords:** Amazon Basin, horseflies, neotropics, taxonomy

## Abstract

*Protosilvius gurupi* **sp. n.** (Tabanidae, Pangoniinae) is described and illustrated based on seven female and 53 male specimens collected in the Amazonian region at Reserva Biológica Gurupi, Centro Novo do Maranhão municipality, northwest Maranhão, Brazil. This is the first record of *Protosilvius* in northern Brazil and in the Amazon Basin. An illustrated key to all *Protosilvius* species is also presented.

## Introduction

Currently, *Protosilvius* Enderlein, 1922 has been recorded only in Brazil. The genus was originally described to include *Protosilvius termitiformis* Enderlein, 1922. Fairchild's (1962) revision synonymized *Histriosilvius* Kröber, 1930 under *Protosilvius*, transferring *Histriosilvius longipalpis* Macquart, 1848 and describing three species, totaling five species, namely: *Protosilvius termitiformis* (type-species), *Protosilvius longipalpis*, *Protosilvius phoeniculus* Fairchild, 1962, *Protosilvius priscus* Fairchild, 1962 and *Protosilvius mackerrasi* Fairchild, 1962. Fairchild (1962) considered *Protosilvius* as part of the more basal tribe Pangoniini and to be closely related to the Australian genus *Ectenopsis* Macquart, 1838 and the Nearctic genera *Apatolestes* Williston, 1885 and *Asaphomyia* Stone, 1953. *Protosilvius* never was included in a phylogenetical analysis and this concept was not corroborated yet.

Enderlein's (1925) type specimen description is very short and insufficient to identify any specimen to species level. Fairchild (1962) re-described *Protosilvius termitiformis* and made a key to all known species at the time. Fairchild and Burger's (1994) catalog reports the following Brazilian records (in parenthesis) for *Protosilvius* species: *Protosilvius longipalpis* (unknown state), *Protosilvius mackerrasi* (São Paulo: Bananal), *Protosilvius phoeniculus* (Rio de Janeiro: Itatiaia), *Protosilvius priscus* (Goiás: Leopoldo Bulhões and Anápolis) and *Protosilvius termitiformis* (Minas Gerais: São João del Rei). Turcatel et al. (2007) extended the geographical record of *Protosilvius termitiformis* to Paraná, Fóz do Iguaçu, south of Brazil. The specimens recorded to Paraná were checked by FLO (junior author) at Universidade Federal do Paraná and they belong to a different genus, so this record was based on misidentified specimens. This study records *Protosilvius gurupi*. sp. n. in Maranhão, the first record in the Amazon Basin.

## Material and methods

This study is based on the examination of 60 specimens collected at Reserva Biológica do Gurupi (Rebio Gurupi) (03°14'05"S, 46°41'83"W) of the Instituto Chico Mendes de Conservação da Biodiversidade (ICMBio), in northwestern Maranhão state, Brazil. Rebio Gurupi is in an Amazonian region composed mainly of primary *terra firme* rainforest. Specimens were collected using a "mobile" light-trap, which consisted of a white sheet (1.2 × 1.2 m) hung vertically and lit by two mercury vapour lamp (160 watts) set in the storage trunk of a pick-up truck (Fig. 28). The truck was moved slowly and continuously (4 km/h) along an unpaved road surrounded by forest, stopping each 200 meters for 30 minutes. Collecting took place from 08:00 pm to 04:00 am by two persons on each side of the light trap (Fig. 28). Specimens that landed on the sheet were collected using a vial with ethyl acetate and brought to the laboratory for sorting, mounting and species identification.

The morphological terminology and figure abbreviations are based on Cumming and Wood (2009). The description was made using a Leica M125 stereoscopic microscope with an incident white light source.

The material collected was deposited in the following institutions: Coleção Zoológica do Maranhão (CZMA), Universidade Estadual do Maranhão, Caxias, Maranhão, Brazil; Instituto Nacional de Pesquisas da Amazônia (INPA), Manaus, Amazonas, Brazil; Museu Paraense Emílio Goeldi (MPEG), Belém, Pará, Brazil; and Museu de Zoologia da Universidade de São Paulo (MZSP), São Paulo, São Paulo, Brazil.

The new species description was based solely on the holotype specimen. The opposite sex, based on paratype specimens, and the variations between individuals are discussed separately. The specimen length was based on the straight distance measured from the frons at antenna level (antenna excluded) to the apex of the abdomen. Wing length is the straight distance measured from the base of the costal vein to the wing apex. Label data are cited in full, including original spelling, enclosed in quotation marks (“”), with punctuation and date transcribed from the top downward. Square brackets ([ ]) are used to indicate information that is not included in the original label. The terminology used follows Cumming and Wood (2009). The new species description was based on same characters used for *Protosilvius termitiformis* re-description (Fairchild 1962) so both descriptions would be comparable considering the updated terminology, e.g. basal plate = postpedicel.

The apex of the abdomen was removed and then macerated in heated 85% lactic acid (Cumming 1992) so the terminalia could be dissected and then examined in an excavated slide with glycerin. Terminalia were then placed in a microvial with glycerin and pinned with their associated specimen. Structures were photographed using a Leica DFC500 digital camera fitted on a Leica MZ205 stereomicroscope and connected to a personal computer with the Leica Application Suite software, which includes an Auto-Montage module (Syncroscopy software) (http://www.syncroscopy.com/syncroscopy/) which produces a composite image of different focal point taken from the specimen. The keys and illustrated figures presented are modified from Fairchild (1962). It should be noted that Fairchild did not insert scale bars, since all species are about the same size, and all figures were reduced in the same proportion.

## Results

### Protosilvius gurupi

Rafael, Marques & Limeira-de-Oliveira sp. n.

urn:lsid:zoobank.org:act:3A1AA834-5114-4918-BC20-514836F16539

http://species-id.net/wiki/Protosilvius_gurupi

Figs 1–11

#### Material.

**HOLOTYPE** female. “Brasil, MA[ranhão] [Centro Novo do Maranhão] REBIO – Res[erva] Biol[ógica do] Gurupi 03°14'05"S, 46°41'83"W “Arm[adilha] Luminosa móvel 07–15.I[Jan.].2011, F. Limeira-de-Oliveira & M. M. Abreu, cols.” (CZMA). Paratypes: same data as holotype (5 females, 22 males, CZMA; 2 females, 20 males, INPA; 5 males, MPEG; 5 males, MZSP).

#### Diagnosis.

Mostly light yellow, slender, and soft-bodied specimens. Thorax and abdomen with yellow bristles. Antenna with three flagellomeres after postpedicel. Wing unusually long; usually with cup cell open, without petiole if cell is closed. Abdomen unicolorous. Female tergite 9 distinctly narrower medially; tergite 10 sub-rectangular**.**

#### Description.

**Figures 1–11.**
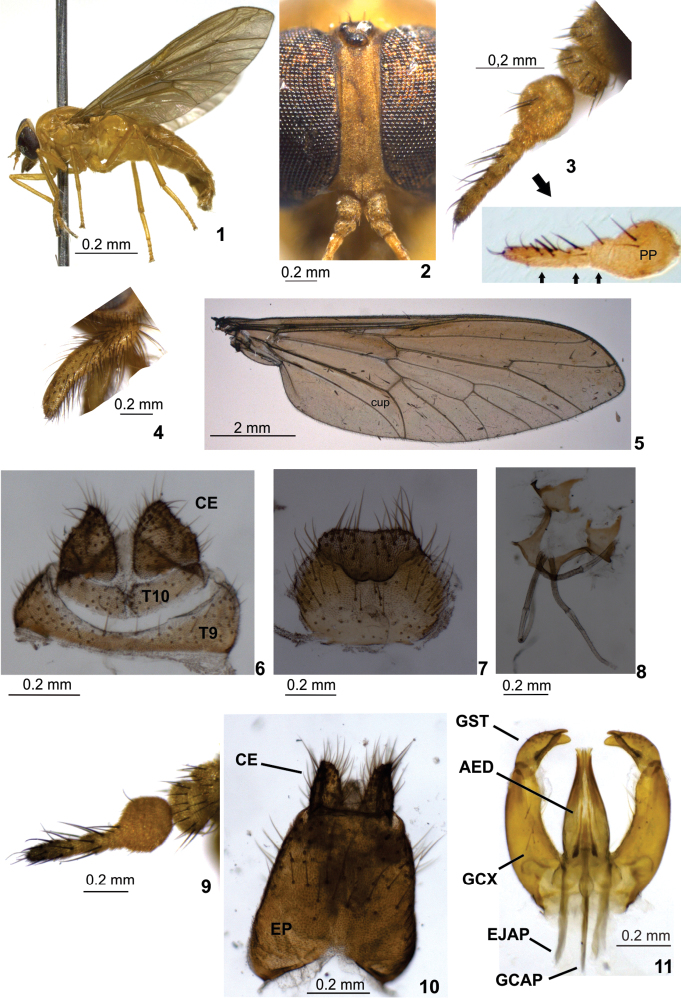
*Protosilvius gurupi*, sp. n., paratype female. **1** habitus **2** frons **3** antenna; below detail of clarified antenna of a different paratype showing sutures between distal flagellomeres (distal flagellomeres indicated by smaller seta) **4** palpus **5** wing **6** tergite 9, tergite 10 and cercus **7** sternite 8 and gonapophysis **8** genital fork and spermathecal ducts **9–11** paratype male **9** antenna **10** epandrium and cercus **11** gonostylus and aedeagus. Figs **1, 3, 4, 9** in lateral view; **2** in frontal view; **5, 6, 8 10** in dorsal view; **7, 11** in ventral view. Abbreviations: **AED** = aedeagus, **CE** = cercus, **EJAP** = ejaculatory apodeme, **EP** = epandrium, **GCAP** = gonocoxal apodeme, **GCX** = gonocoxite, **GST** = gonostylus, **PP** = postpedicel, **T** = tergite.

Holotype female. Body length: 8.9 mm. Specimen mostly light yellow. Head (Fig. 1) with eyes black (green in life,) more or less suboval in profile, rounded laterally in frontal view, with very short yellowish bristles which are barely visible under higher magnification. Frons (Fig. 2) narrow, somewhat parallel sided, slightly divergent dorsally and ventrally, frontal index about 2.7, smoothly tomentose, with a median inconspicuous groove, and short, inconspicuous brown bristles. Ocellar tubercle (Fig. 2) somewhat prominent, as high as ocellus. Subcallus (Fig. 2) very small, tomentose, separation from frons indistinct. Parafacial narrow, tomentose, with long black bristles. Face convex laterally, deeply sunken medially, tomentose, without bristles, separate from parafacial by deep groove. Antenna (Fig. 3) with scape and pedicel short, plump, yellow to brown, and with robust black bristles; flagellum light yellow with robust black bristles, apparently with six flagellomeres; postpedicel swollen when observed in lateral view, with three distal flagellomeres, the first flagellomere almost totally fused to postpedicel based on a distinct incomplete suture on medial side (see Fig. 3 from a clarified antenna of a different paratype specimen); second flagellomere as long as first and with an indistinct suture; third flagellomere, the distalmost, longer than two preceding flagellomeres. Palpus (Fig. 4) with first segment somewhat swollen, second slightly narrower and slightly curved, distinctly bristled. Proboscis short, as long as palpi, membranous, with long, narrow, soft and bristled labellum.

Thorax with scutum and scutellum light brown to dark yellow, sparsely yellow bristled, with yellow pruinescence. Pleuron slightly clearer than scutum, yellow with light grey to yellow pruinescence.

Legs (Fig. 1) entirely yellow except distal half of tarsomeres 5 brown; most legs with yellow bristles, except fore tibia black bristled. All tarsomeres 1 of equal length. Hind tibial spurs slightly shorter than mid ones.

Wing (Fig. 5) 9.1 mm long, 2.9 mm wide, narrower than usual for tabanids, diffusely brownish, with costal margin slightly darker; pterostigma ill defined. Vein Sc bare dorsally and ventrally; vein R_4_ with short appendix; vein CuA_1_ with even row of small setulae; cell cup open. Halteres with stem yellow and capitulum brown and white.

Abdomen (Fig. 1) long, narrow, entirely yellow, with short golden bristles dorsally and ventrally. Terminalia: Tergite 9 (Fig. 6) narrow medially, expanded laterally; tergite 10 subrectangular in dorsal view, divided medially; cercus subtriangular. Sternite 8 (Fig. 7) wider than long, with somewhat distinct gonapophysis. Genital fork as in figure 8.

**Male**. Body length: 9.0 mm; wing length: 9.1 mm. Habitus similar to female specimens except head holoptic, antenna (Fig. 9) slightly weaker, cell cup narrowly open (sometimes narrowly closed, without petiole), abdomen slender and of a lighter tone, first 3–4 abdominal segments light yellow, somewhat translucent, remaining brown. Terminalia (Fig. 10): epandrium with concavity basally; cercus subquadrate in lateral view; gonocoxite slightly arched; gonostylus bifid (Fig. 11); ejaculatory apodeme and gonocoxal apodeme similar in length.

#### Etymology.

The specific epithet is a noun in apposition and refers to Reserva Biológica do Gurupi, where the specimens were collected.

#### Distribution.

Brazil, Maranhão.

#### Holotype condition.

Pinned, not dissected, in good condition except for a damaged left wing. We chose the best preserved specimen, among the few females collected, as holotype because in most tabanids species the primary types are females.

#### Variation.

One female specimen without short appendix on vein R_4_. Female size varying from 8.6–9.6, mean 9.0 mm (n = 3). Male size varying from 8.0–10 mm, mean 9.1 cm (n = 10).

#### Discussion.

*Protosilvius gurupi* sp. n. is smaller than other *Protosilvius* species, as the biggest specimens (9.8 mm) are slightly shorter than the smallest species, *Protosilvius priscus* (10 mm); these differ by three flagellomeres after the postpedicel in the former and four flagellomeres in the latter. Female specimens would key out to *Protosilvius termitiformis* in couplet 3 of Fairchild´s (1962) key by the following characters: short and sparse bristled specimens and abdomen unicolorous. *Protosilvius gurupi* has an open cup cell and narrow female tergite 9 (Fig. 6), whereas *Protosilvius termitiformis* has a closed cup cell and wide female tergite 9 (Fig. 20). According to Chainey and Hall (1996), female specimens of *Protosilvius* differ from *Boliviamyia* Chainey & Hall by a frons without callus, a slender palpus without a dorsal groove and apparently absent mandibles and both sexes have the antennal flagellum with a very short and/or irregular postpedicel and very long and slender apical flagellomeres.

Bionomics. Light traps are a common method for collecting many male and some female tabanids. All specimens of both sexes of *Protosilvius gurupi* sp. n. were collected in light traps, not one in the Malaise traps mounted nearby. The specimens were constantly collected in the light trap, either while the car was slowly moving or not. We believe the specimens are not nocturnal but they were attracted to trap when the light reached the specimens bedding in the vegetation. The collection was made in the Amazonian Region, in the state of Maranhão, in the rainy season, far from any drier area for at least 300 kilometers.

### Key to female specimens of *Protosilvius*

**Table:** 

1	Frons distinctly widening dorsally and ventrally (Fig. 12), over 4× as high as narrowest width. Flagellum with rather elongate postpedicel and distal portion 4-segmented, distal flagellomere longer than three preceding ones (Fig. 13). Abdominal tergites banded with silvery-white bristles on posterior margin	*Protosilvius longipalpis*
–	Frons narrow, nearly parallel sided, divergent ventrally or slightly wider dorsally and ventrally. Flagellum not as above. Abdominal tergites with only black bristles or banded with yellowish bristles on posterior margin	2
2	Frons divergent ventrally (Figs 21, 24)	3
–	Frons parallel sided (Fig. 14) or slightly divergent dorsally and ventrally (Figs 2, 18)	4
3	Frons around 3× as high as narrowest width just below ocelli (Fig. 21). Distal flagellomere longer than three preceding flagellomeres (Fig. 22)	*Protosilvius priscus*
–	Frons over 4× as high as narrowest width just below ocelli (Fig. 24). Distal flagellomere of similar length to preceding flagellomeres (Fig. 25)	*Protosilvius mackerrasi*
4	Frons parallel sided (Fig. 14), less than 3× as high as dorsal width, just below ocelli. Postpedicel divided into 3 flagellomeres with another partial division, so that the flagellum may seem incompletely 8–segmented (Fig. 15). Scutum and scutellum black bristled. Abdominal tergites with band on posterior margin formed by yellow bristles	*Protosilvius phoeniculus*
–	Frons somewhat parallel sided to slightly divergent dorsally and ventrally (Figs 2, 18), more than 3× as high as dorsal width, just below ocelli. Postpedicel with flagellomeres somewhat fused (Figs 3, 19). Scutum and scutellum yellow bristled. Abdominal tergites without band on posterior margin, bristles unicolorous	5
5	Cell cup closed, with short petiole. Tergite 9 uniformly wide medially and laterally, and tergite 10 wider medially in dorsal view (Fig. 20)	*Protosilvius termitiformis*
–	Cell cup open (Fig. 5), if closed then without petiole. Tergite 9 distinctly narrower medially and tergite 10 somewhat rectangular (Fig. 6) in dorsal view	*Protosilvius gurupi* sp. n.

### Key to male specimens of *Protosilvius* (*Protosilvius termitiformis* and *Protosilvius longipalpis* are not included in this key because males are unknown)

**Table:** 

1	Distal flagellomere widened (Fig. 16). Abdominal tergites with band on posterior margin formed by yellow bristles. Gonostylus with swollen base and bifid appendages ventrally directed (Fig. 17)	*Protosilvius phoeniculus*
–	Distal flagellomere not widened (Figs 9, 26). Abdominal tergites without band on posterior margin, bristles unicolorous. Gonostylus base not swollen and bifid appendages medially directed (Figs 11, 23, 27)	2
2	Upper eye facets enlarged. Medial margin of gonocoxite nearly straight and phallus ends at level of gonocoxite apex (Fig. 23)	*Protosilvius priscus*
–	Upper eye facets not enlarged. Medial margin of gonocoxite slightly curved and phallus apex ends after gonocoxite apex (Figs 11, 27)	3
3	Blackish specimens with blackish wings	*Protosilvius mackerrasi*
–	Yellowish specimens with diffusely brownish wings (as in figure 5)	*Protosilvius gurupi* sp. n.

**Figures 12–27.**
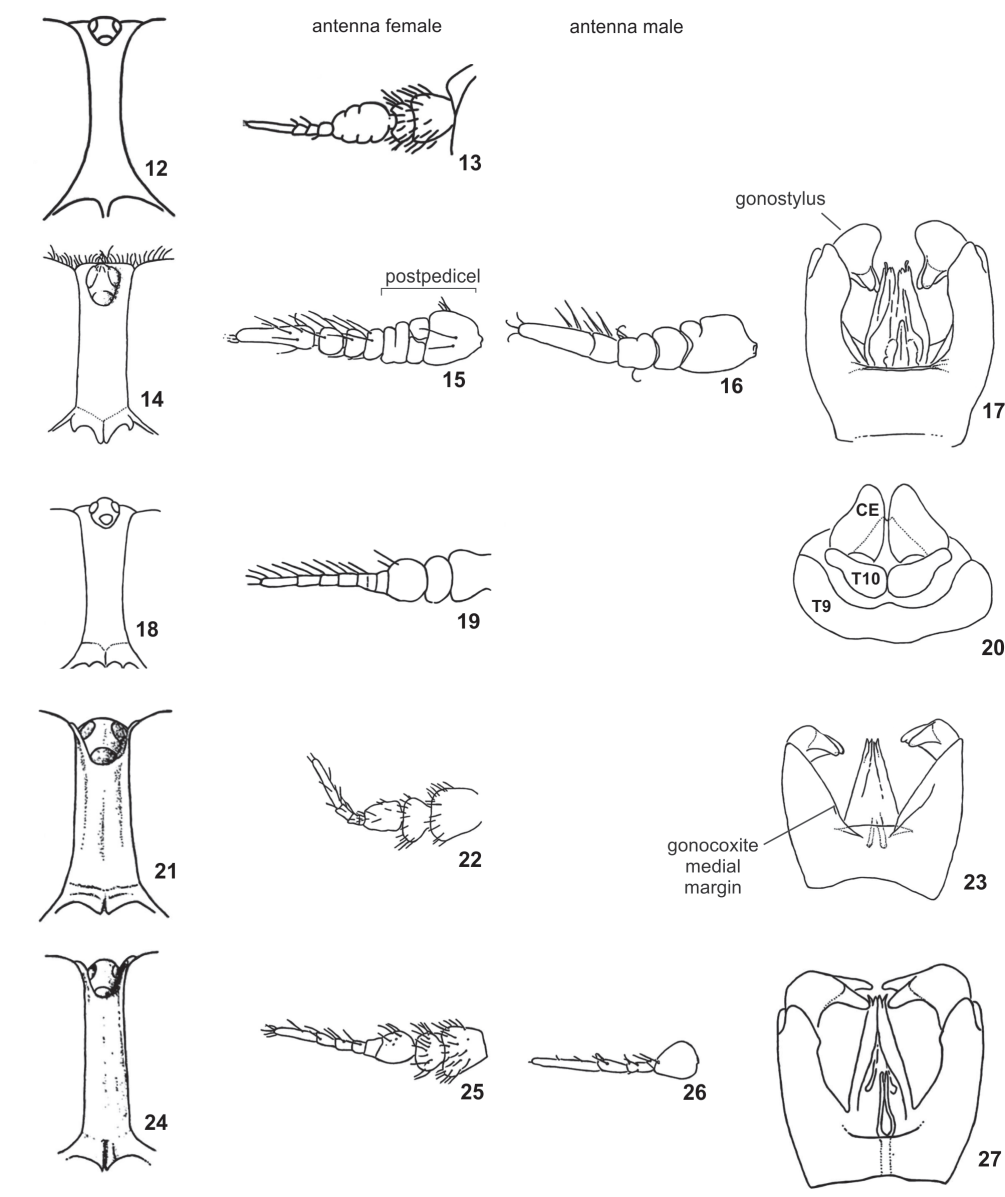
*Protosilvius* figures from Fairchild (1962). **12–13** *Protosilvius longipalpis*;**12** frons **13** antenna, female **14–17** *Protosilvius phoeniculus* **14** frons **15** antenna, female **16** antenna, male **17** genitalia, male **18–20** *Protosilvius termitiformis* **18** frons **19** antenna, female **20** tergite 9, tergite 10 and cercus **21–23** *Protosilvius priscus* **21** frons **22** antenna, female **23** genitalia, male **24–27** *Protosilvius mackerrasi* **24** frons **25** antenna, female **26** antenna, male **27** genitalia, male. Figs **12, 14, 18, 21, 24** in frontal view; **13, 15, 16, 19, 22, 25, 26** in lateral view, all arranged in the same orientation, **17, 20, 23, 27** in dorsal view; **7, 11** in ventral view. Abbreviations: **CE** = cercus, **T** = tergite.

**Figure 28.**
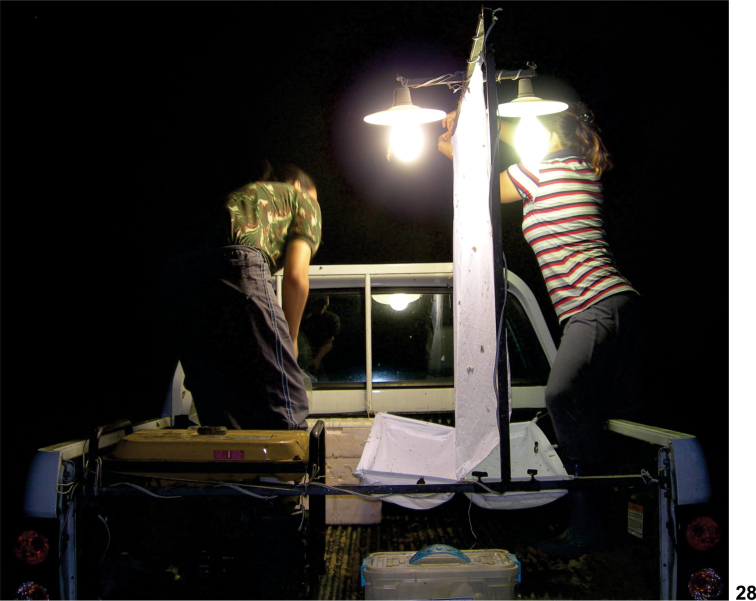
“mobile”light trap placed on a pick-up truck.

## Supplementary Material

XML Treatment for Protosilvius gurupi
